# PortAqua: a low-cost, compact water quality meter for science communication

**DOI:** 10.1007/s10661-022-10804-3

**Published:** 2023-01-27

**Authors:** Carlos A. Pérez-López, Wendy Pantoja, Jaime A. Pérez-Taborda, Alba Ávila

**Affiliations:** 1 Centro de Microelectrónica (CMUA), Departamento de Ingeniería Eléctrica y Electrónica, Universidad de los Andes, Carrera 1 No. 18A - 12, Bogotá, 111711 Distrito Capital Colombia; 2 Grupo de Nanoestructuras y Física Aplicada (NANOUPAR), Escuela de pregrados-Dirección Académica, Universidad Nacional de Colombia Sede De La Paz, Km 9 vía Valledupar La Paz, La Paz, Carrera 1 No. 18A - 12 Cesar Colombia

**Keywords:** Water quality, Low-cost water meter, Additive manufacturing, Educational electronics

## Abstract

Water quality monitoring allows communities to achieve sustainable management of water resources, which is crucial for life-supporting processes. Water quality is determined by measuring chemical, physical, and biological parameters, requiring sophisticated meters and trained specialists to perform the measurement. However, in low-income communities, water quality is determined by using human senses—smell, color, and taste—since meter acquisition is limited by costs and most people do not know how to monitor water quality. Therefore, accessible technology is necessary to empower communities to have a sustainable lifestyle. In this paper, we present the design and implementation of PortAqua, a 2-parameter water quality meter (WQM), to promote training on water quality measurement. Using basic electronic components, PortAqua is capable of measuring pH with an error of 0.4, and conductivity with an error of 33% at 85 µS cm^-1^, and 8.7% at 1413 µS cm^-1^. To demonstrate its preliminary effectiveness as a WQM and its science communication capabilities, the meter has been used in a hands-on workshop with undergraduate and graduate students. During the workshop, attendees participated in a short lecture about water quality measurement techniques and local regulations. Then, they collected water samples from a local source, measured the samples using PortAqua, and discussed the results based on the concepts and regulations. The workshop’s effectiveness was evaluated through pre- and post-assessments which revealed increased knowledge of water quality regulations, measurement, and parameters at the end of the activity.

## Introduction

The use of technological tools in educational environments has been shown to have a positive impact on increasing the student learning process Ekin et al. (2021); Zhang et al. (2012); Allam et al. (2014). Some of these tools allow students to interact with the environment by measuring physical or chemical variables, thus improving their understanding of difficult concepts Ekin et al. (2021). In addition, the development of accessible technological tools has been used to teach non-expert communities about the importance of monitoring their environment to identify changes that can affect their health. Determining water quality is crucial to ensure human health. This is especially important for communities from Colombia, which have limited access to clean water. The Colombian Caribbean San Andres Island has notable difficulties in obtaining drinkable water. Carolina Sofia Velásquez Calderón (2020). In Bogota, D.C., the river that crosses the city has unacceptable water quality; only five from twenty-nine water quality measurement stations have shown acceptable quality levels Díaz-Casallas et al. (2019). In the Cauca region, the Cauca River is highly susceptible to increased water pollution events, which can saturate the water cleaning facilities and force them to shut down. Vélez et al. (2014). While this information is openly available, the generalized low level of literacy in measuring water quality among undergraduate students results in low perception of these problems and low action to correct it Johnson and Courter (2020). Measuring water quality could be challenging for a community of laypeople without adequate tools. To address this issue, accessible technological tools are an option to facilitate the understanding of water quality parameters Johnson and Courter (2020); Burrows et al. (2018); Petrov et al. (2021).

Currently, to determine water quality, biological, physical, and chemical characteristics are measured United States Enviromental Protection Agency Office of Water (2017); REPÚBLICA DE COLOMBIA (2007). These measurements are performed through several techniques, including laboratory tests and water quality meter (WQM) that can be single or multi-parameter Amer and Mohamed (2022). Two water quality parameters are the potential of hydrogen (pH) and conductivity ($\sigma$) Hassan Omer (2020). pH is an indicator to determine the acidity or alkalinity of a substance. $\sigma$ is used to establish salinity level, and previous studies have shown their correlation with total dissolved solids. Therefore, monitoring the pH and the $\sigma$ could be the first approach to detect changes in water quality and generate an early warning for advanced water quality measurement techniques Rusydi (2018).

At present, there is an increasing interest in developing low-cost WQMs to offer a more cost-effective technology for zones where the quality of the water is critically compromised Bernalte et al. (2020), and ensure that drinking water is free from pollutants that could cause health issues. Recent monitoring systems are as follows: remote robots based on Internet of Things Adhipramana et al. (2020); Chowdury et al. (2019), real-time measurement system Pasika and Gandla (2020), and wireless communication Alam et al. (2021). A compelling application of WQM has been implemented in the Cauca River located in Colombia. This is used as an early alert for identifying contaminants and pollutants in the water that could saturate the local water processing system and affect the population Vélez et al. (2014). Although these systems have shown reliable water quality parameter measurements, the feasibility of using the system for science communication has not been evaluated. Increasing access to safe water requires citizens to learn the fundamentals of water quality parameters monitoring. This is necessary for them to be able to make appropriate decisions about the use and consumption of water

In this work, we propose a portable and low-cost two-parameter water quality meter called PortAqua. This meter can measure pH and $\sigma$ using easy-to-find electronic components. The meter integrates an OLED screen and two buttons that provide the user interface. In addition, a transparent package was designed to enable the user to observe the electronic components without disassembling the meter. The meter was calibrated and tested against commercial calibration buffers and compared with different commercial water quality meters to validate the data measurement.

The main outcomes of PortAqua are:

1. *Precise:* pH measurement on PortAqua shows an error of up to 0.4 units, while conductivity measurement errors were 33% at 85 µS cm^-1^ and 8.7% at 1413 µS cm^-1^.
2. *Portable:* The meter includes a battery that allows for up 8 h of continuous operation. Moreover, its dimensions ($12\times 3.5\times 2$ cm) and low weight (110 g) allow for single-handed operation.
3. *Flexible calibration:* PortAqua can use any parameter standard solution for its calibration and is not limited in calibration points, allowing the use of custom standards and improvements in the measurement. The meter calibration can be performed in two ways. In the first, the meter guides the user on a step-by-step basis using the OLED screen module and using three pH (4.01, 7.01, and 10.01) and conductivity (0, 85, and 1413 µS/cm) standards. This is useful for laypersons or occasional users with little training. The second allows for the modification of the buffer standards and adding more points to the calibration regression calculation, but it requires modifying the text file containing the calibration parameters and uploading it to the meter.
4. *Engaging:* The meter comes in a transparent case that allows the user to see the internal components and correlate parameter measurement concepts to the implementation.
5. *Open source:* Both hardware (PCB, bill of materials, and 3D printing files) and firmware are available in an open repository for anyone that wants to build or work on PortAqua. Perez-Lopez et al. (2021).
6. *Easy to source and affordable:* The cost of the meter’s components is under $\text {US}\$50$ (data from December 2021) and they are available in different non-specialized global marketplaces.
7. *Easy to assemble:* PortAqua’s case can be printed on any printer using the provided 3D files.

The PortAqua meter was used in a hands-On workshop with undergraduate and graduate students to introduce water quality concepts and measurement techniques. The workshop included three stages: (1) preliminary assessment and contextualization, (2) observation and sample collecting, and (3) measurement of samples and final assessment. The preliminary and final assessment allows for the measurement of the effects of the experience. As a result, the students who attended the workshop learned about water quality parameters, local regulations, and the measurement techniques used in PortAqua. They were also more conscious about the importance of supporting their criteria with data. This result suggests that accessible technologies in the educational field can facilitate the learning process and promote continued student participation and interaction.

## Materials and methods

### The meter

**Fig. 1:**
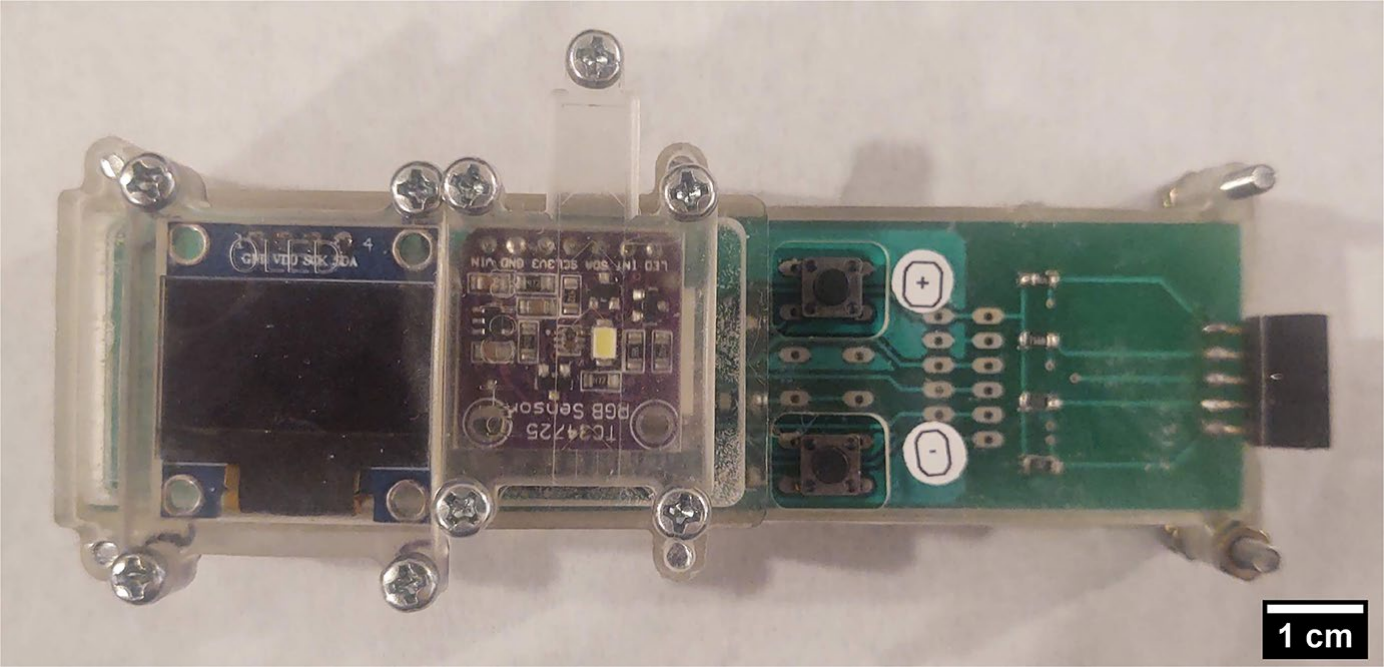
Fully assembled PortAqua 2-parameter water quality meter

**Fig. 2:**
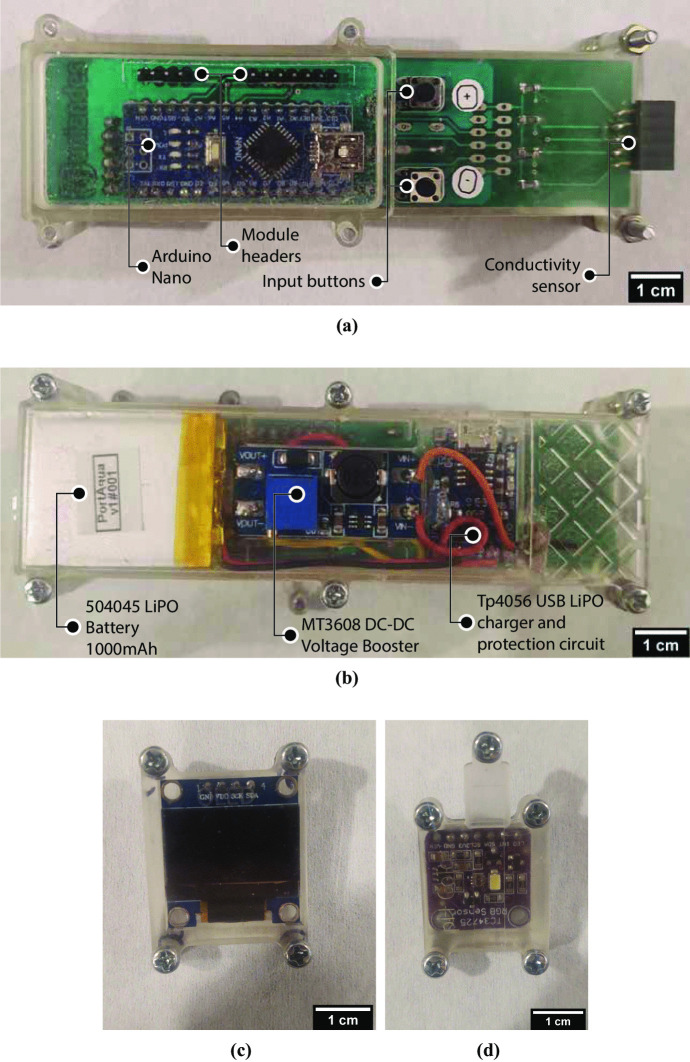
PortAqua modular components: **a** front and **b** back of the main module, components are indicated in labels. **c** OLED interface module, and **d** pH color measurement module

**Fig. 3:**
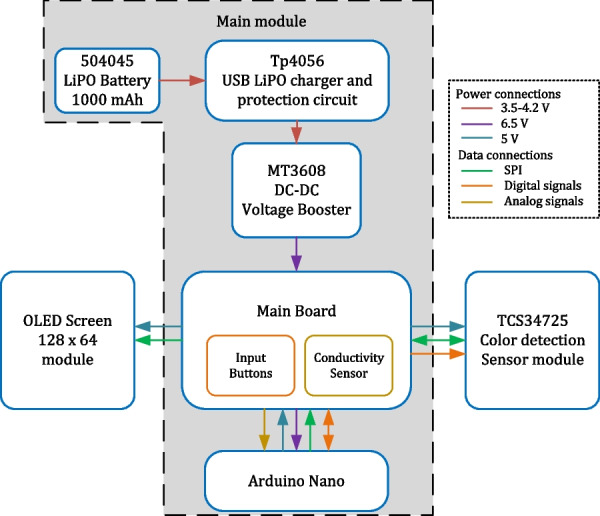
Block diagram of PortAqua: Each block correspond to a component in the meter; the gray section corresponds to the main module. The legend describes the connection type between components

The PortAqua 2-parameter WQM is a handheld meter for measuring the pH and $\sigma$ of water (Fig. 1). It measures $12\times 3.5\times 2$ cm when fully assembled, and weighs approximately 110 g which makes it comfortable for single-hand operation while using the other hand to hold water samples. The meter is composed of a group of modules (see photos in Fig. 2 and the block diagram in Fig. 3). The main module integrates a 504045 LiPO battery (rechargeable single cell, 3.7 V, 1000 mAh) and a TP4056 USB LiPO charger. The battery voltage is boosted to 6 V by a MT3608 Booster to power the Arduino board. The Arduino regulates the voltage to 5 V to power the connected modules. The meter also includes an OLED screen ($128\times 64$) with a SPI connection and two push buttons labeled + and - connected to each digital pin. The parameter measurement modules for pH and $\sigma$ are described.

#### pH measurement

pH is estimated using a TCS34725 color sensor that detects the four Merck ColorpHast pH strip indicators and compares it to a table containing the calibration values for multiple pH levels. The resulting value of the color sensing measurement is an array of three float values representing the *R*, *G*, and *B* values. To compare the calibration values $(V_c)$ in the table to the measured values $(V_m)$, a tri-dimensional space is superimposed, and the spatial distance (*d*) is calculated by using the Euclidean norm between the difference of $V_c$ and $V_m$:

1 $$\begin{aligned} \begin{aligned} V_c&=\begin{bmatrix}R_c&G_c&B_c\end{bmatrix}\\ V_m&=\begin{bmatrix}R_m&G_m&B_m\end{bmatrix}\\ d_i&=\begin{Vmatrix}V_c-{V_m}_i\end{Vmatrix}\\ d_i&=\sqrt{(R_c-{R_m}_i)^2+(G_c-{G_m}_i)^2+(B_c-{B_m}_i)^2} \end{aligned} \end{aligned}$$

Here, the *i* sub-index indicates the calibration standards stored in the microcontroller memory; three pH buffers of 4.01, 7.01, and 10.01 (corresponding to commercial calibration standards) are selected. Next, the local minimum—first $(A_j)$ and second $(B_k)$ of the distances—is selected, and then the pH value is estimated from a linear estimation between both points which are:

2 $$\begin{aligned} \begin{aligned} A_j&=\min _1({d_j}|_{j=1}^3)\\ \end{aligned} \end{aligned}$$

3 $$\begin{aligned} \begin{aligned} \text {pH}&= \begin{matrix} j+\frac{|j-k|}{A_j+B_k}A_j &{} \text {if } i \le j \\ k+\frac{|j-k|}{A_j+B_k}B_k &{} \text {if } i > j\end{matrix} \end{aligned} \end{aligned}$$

The color detection on pH measuring strips results in an objective measurement regardless of the color identification capabilities of the user or light availability. Moreover, it allows for increased precision between pH levels.

#### Conductivity measurement

**Fig. 4:**
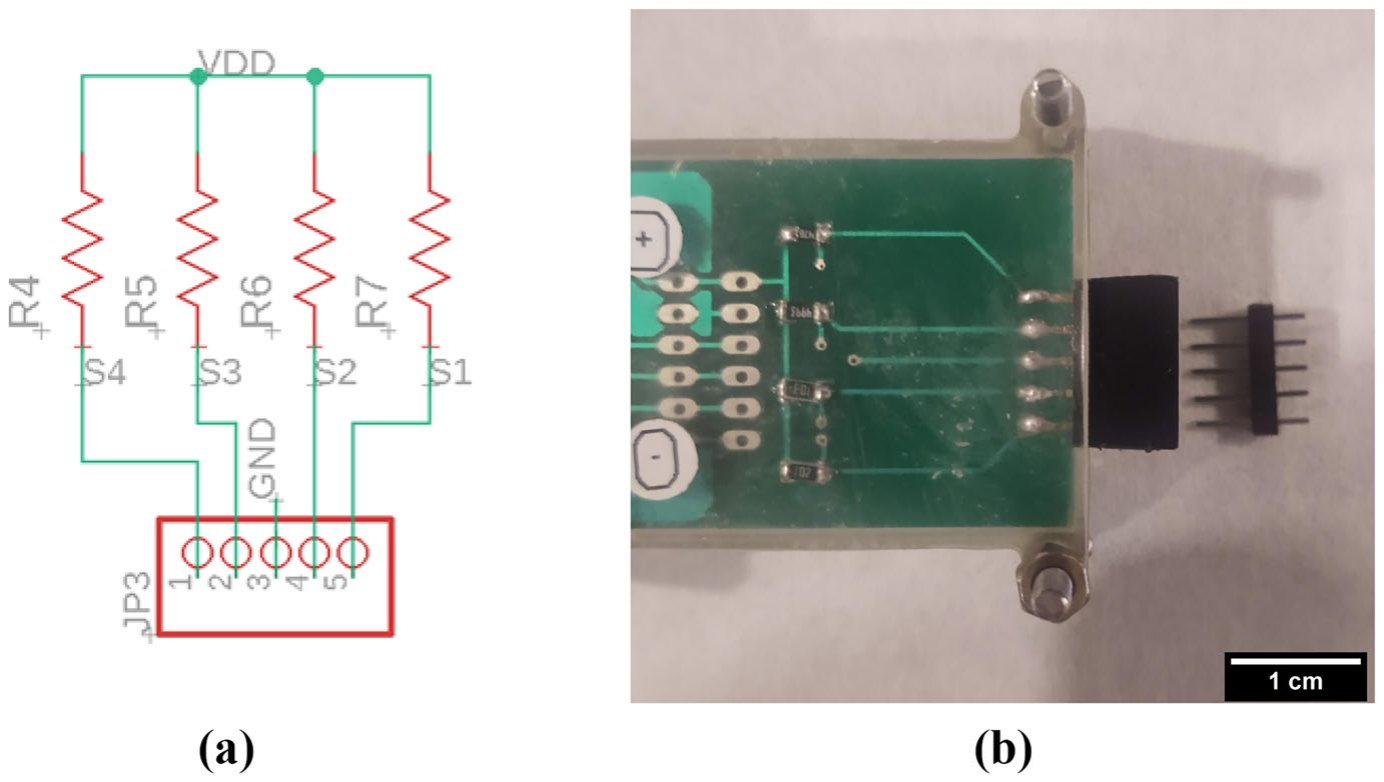
Conductivity meter voltage divider array: **a** schematic and **b** PCB with disposable electrode. The resistances $R_4$ to $R_7$ correspond to 1 M$\Omega$, 100 k$\Omega$, 10 k$\Omega$, and 1 k$\Omega$, respectively

Water conductivity is measured by means of a voltage divider array. Male header pins are used as disposable sensors connected to a 5-V source in the Arduino through a set of resistors. The circuit divides the voltage between the known resistors and the measured water that connects to the ground pin (see Fig. 4). When a sample is measured, the 10-bit ADC on the Arduino maps the analog measurements ($\text {S}_1$ to $\text {S}_4$) to a digital value. Then the closest from the four measurements to 512 bits is used to calculate the conductivity values by applying a conversion curve obtained by linear regression of measurements of commercial conductivity calibration standards at values of 85 µS cm^-1^, and 4515 µS cm^-1^.

### Hands-on community workshop

The hands-on workshop was proposed as a three-stage activity that involved the academic community of Universidad de los Andes in Bogota, Colombia. The workshop was attended by seven undergraduate students and eight graduate students aged between 20 and 35. The first and third stages were performed at engineering laboratories. The second stage was carried out at a local water source called the San Francisco River (SFR) and its small canal known as *Eje ambiental*.

In the first stage, the participants were given a preliminary evaluation to determine their base knowledge level. After this preliminary evaluation, the Colombian standard for regulating water quality was explained. This explanation was designed to give feedback to the previous questions and teach students the ranges accepted for pH and conductivity In addition the most common measurement techniques were introduced.

**Fig. 5:**
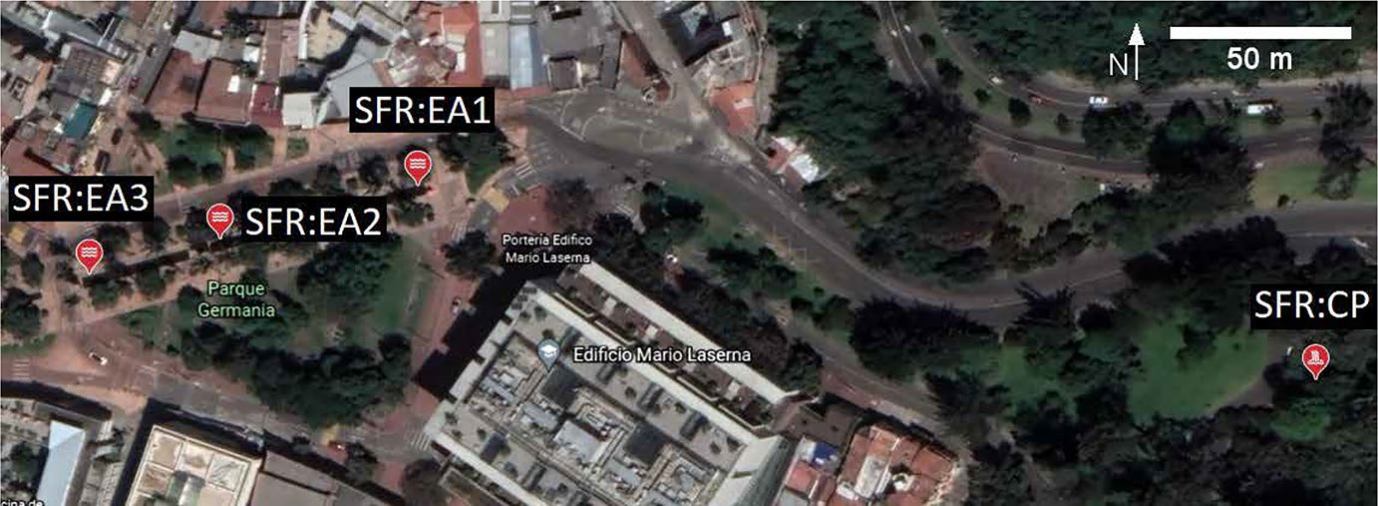
Hands-on workshop water collection locations. The map shows the San Franciscto River and its canalized region around Universidad de los Andes Campus, Bogota, Colombia (lat 4,6029004, lon −74,0650599). The labels correspond to measurement locations: (SFR:CP) is the canalization point of the river, and (SFR:EA1 to EA3) are points of its urban canalization known as *Eje Ambiental*

In the second stage, the group collected water samples at three different SFR sites (see Fig. 5), where they made observations about the river and the economic and community activities connected to the water resources, and reflected on their environmental effect and impact.

In the third stage, one of the organizers introduced the water quality analysis techniques and Colombian regulations. Attendees were allowed to use the PortAqua devices on the samples taken from SFR and local potable sources. The results of the hands-on workshop show the feasibility of knowledge transfer to communities using PortAqua. The students were able to identify samples that are within acceptable values for the pH and conductivity parameters, in accordance with Colombian water legislation.

Finally, students had 15 min to answer an assessment. It included the same questions to quantify changes in knowledge level. To conclude, students gave us feedback about the activity and their perception using the PortAqua.

## Results and discussion

### PortAqua 2-parameter water quality meter

#### pH measurement

**Fig. 6:**
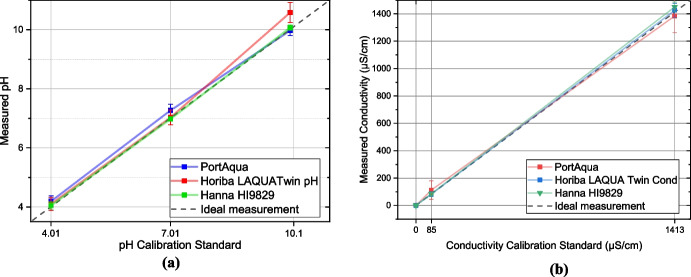
Measurements of (**a**) pH and (**b**) conductivity using PortAqua (Blue) and two commercial water probes: Horiba LAQUATwin (Red) and Hanna HI9829 Multiparameter water quality probe (Green). The measured samples correspond to to Hanna pH calibration buffers kit (4.01, 7.01, and 10.1) and conductivity standards (84 µS cm^-1^, and 1413 µS cm^-1^). The dashed line (black) corresponds to the ideal measurement trend, the glyphs and error bar correspond to the mean value and standard deviation from 5 measurements

After the calibration, the pH lookup table was adjusted according to the calibration standard values. To validate PortAqua measurements, two commercial water meters were used. First, a single-parameter pH meter (Horiba LAQUATwin pH-11) was calibrated using its buffers, 4 and 7. Second, a multi-parameter meter (Hanna HI9829) was calibrated using its buffers, 4, 7, and 10. Three buffers were measured five times using PortAqua and these two commercial water meters (see Fig. 6a).

The results indicate that the PortAqua measurements align with the ideal measurement line at the 4 and 10 calibration standards. In addition, at point 7, the meter shows a higher value than commercial meters. The Horiba meter resulted in the closest measurement with a similar standard deviation for the 4 and 7 pH. However, the measurement at 10 shows a higher standard deviation of 0.7, which can be caused by a lack of a third calibration point.

#### Conductivity measurement

After calibration, the conversion curve equations are adjusted to the values of the conductivity standards, and the measurements were repeated to ensure correct calibration (see Fig. 6b). Three standard samples were measured using PortAqua and two commercial water meters for comparison; a single-parameter conductivity meter (Horiba LAQUATwin Cond-11) and a multi-parameter meter (Hanna HI9829). Each meter was calibrated using its own set of standards, 1411 µS cm^-1^ and 12900µS cm^-1^ for the Horiba meter and 85 µS cm^-1^, and 1413 µS cm^-1^ for the Hanna meter.

Conductivity measurements of PortAqua follow the ideal measurement trend. At a low conductivity of 85 µS cm^-1^, the meter shows a mean error of 33% (22 µS cm^-1^), and at higher concentrations of 1413 µS cm^-1^ the error is reduced to 8.7% (123.69 µS cm^-1^). In comparison to commercial meters, which in all cases the mean error is inferior to 10%, the variance of the PortAqua measurements is approximately 1.5 to 3 times the variance of commercial meters.

#### Current consumption and battery duration

**Fig. 7:**
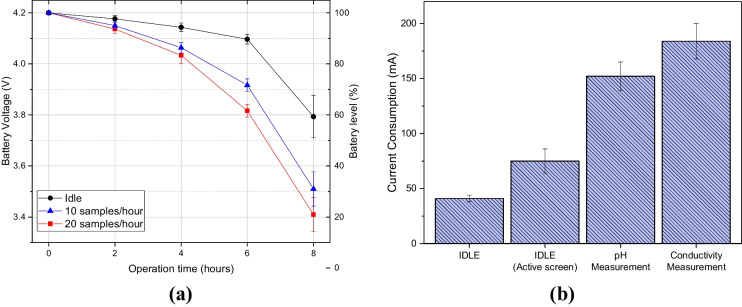
Battery consumption of PortAqua: **a** discharge rate for three different usage trends: no measurements with active screen (black), 10 measurements per hour (blue), 20 measurements per hour (red). Glyph position and error bar correspond to mean value and standard deviation from 3 different PortAqua devices. **b** Current consumption of PortAqua in 4 stages of operation: idle (screen off), idle (active screen), pH measurement, and conductivity measurement. Measurement values correspond to the mean and standard deviation of 30 s of operation in the corresponding stage

PortAqua battery duration was measured to identify the discharge rate. To do this, the voltage level of the single-cell battery was measured (see Fig. 7a). The battery has a maximum voltage of 4.2 V and a minimum of 3.4 V which are considered to be 100% and 0% charge, respectively.

Battery level was measured at intervals of 1 h of operation under three scenarios: (1) idle for no measurements with 20% of active pixels on the OLED screen, (2) for ten measurements per hour, and (3) for 20 measurements per hour. The measurements show that the meter can operate for over 8 h and perform up to 160 measurements of pH and $\sigma$.

The current consumption of the meter was also measured for four operation stages (see Fig. 7b). The stages studied were idle with inactive screen, idle with 20% of active pixels, measuring pH, and measuring conductivity. PortAqua was set up to keep the measured state for 30 s.

The measured values indicate that the measurement stages result in higher consumption than idle, the conductivity measurement being the most exigent. This is caused by the resistance array that consumes a larger percentage of current when calculating the conductivity of the sample.

Compared to the Hanna and Horiba WQMs, PortAqua uses a single-cell LiPO battery which can be recharged via USB though the TP4056 charger. The Hanna meter uses 4 D-type rechargeable NiMH batteries that can be recharged using a proprietary connector and AC adapter and can make over 180 measurements with one charge. The Horiba LAQUATwin meters use a pair of non-rechargeable lithium coin CR2032 batteries which can perform 92 measurements until activation of the low battery indicator. Thus, the PortAqua allows not only flexible operation and recharge, but also it can be recharged by any off-the-shelf USB power adapter or power bank to extend its operation. Moreover, LiPO batteries perform better than NiMH types in terms of efficiency and energy density Kang et al. (2014).

### Community hands-on workshop

**Fig. 8:**
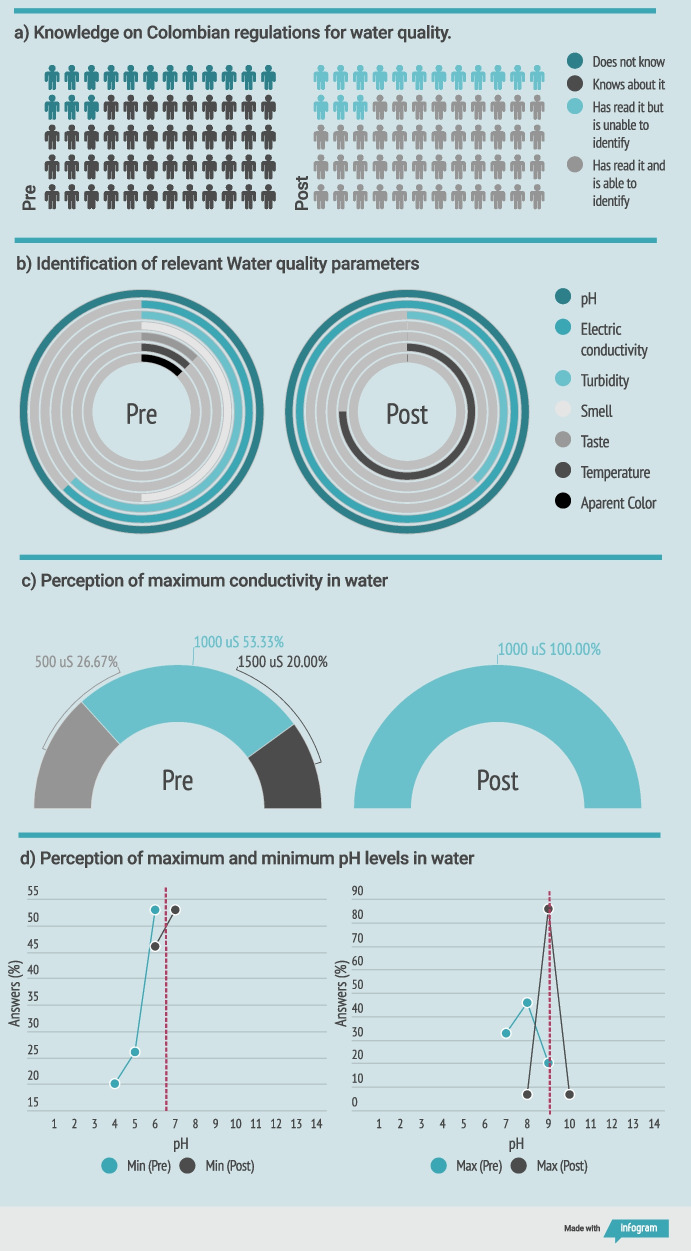
Hands-on workshop assessment results (previous in left, posterior in the right): **a** Colombian regulation knowledge, **b** water quality parameters, **c** maximum conductivity, **d** minimum and maximum pH

The impact of the hands-on workshop was measured by comparing two assessments, a pre-survey administered before the activity, and a post-survey completed immediately after the activity. This comparison makes it possible to determine the knowledge acquired by considering the prior knowledge of the participants. An overview of the community answers is shown in Fig. 8. Pre- and post-assessments have been implemented by Ekin et al. (2021), showing that they are a useful tool to detect misconceptions and to get a general idea of the participants’ familiarity with the subject matter.

Figure 8a shows that before the activity, students were not familiar with the Colombian standard. Nevertheless, the workshop was successful in introducing the standard and the accepted ranges of pH and conductivity. Figure 8b illustrates the relevant water quality parameters identified by the students. We observed that the pH was identified by 100% of the students from the beginning of the workshop, while conductivity was only identified by 62% of the students. After the pedagogic activity, 100% of the students understood that conductivity is an indirect parameter for measuring water quality. Figure 8c presents the students’ answers about what is the maximum conductivity accepted by the Colombian standard for drinking water. Before the activity, students had different opinions, which included values of 500, 1000, and 1500 µS cm^-1^. In the last assessment, 100% of the students answered correctly (1000 µS cm^-1^). Figure 8d shows the students’ answers about what is the minimum and maximum pH accepted by the Colombian standard for drinking water. At the beginning of the workshop, the mean of the answers was under the actual value for both the minimum and maximum pH accepted. After the workshop, the mean was at the correct minimum and maximum pH value of the drinking water.

The results of the hands-on workshop show the feasibility of knowledge transfer to communities using PortAqua. The students were able to identify samples that are within acceptable values for the pH and conductivity parameters set by Colombian water legislation. This is a first step for bringing communities closer to learning the fundamentals of water quality parameters monitoring. With the right fundamentals, citizens can make appropriate decisions about the use and consumption of water. Communities could frequently monitor water pH and conductivity, identifying significant changes in the values that demand a complete water quality analysis. Additionally, involving communities in the measurement of water quality parameters could empower citizens and encourage care for water resources Setälä et al. (2022).

In the feedback session at the end of the workshop, the students expressed interest in the water quality measurements and curiosity for the water quality of the sample neighboring their homes. Moreover, they expressed opinions and ideas about the electronic design, the measurement techniques, and the future expansion of PortAqua. This experience has resulted in stress-free environments where students are learning based on observing things around them and experimenting on it, encouraging them to be involved in the learning process, which improves understanding and retention of concepts, as opposed to just reading or listening Admane and Mondhe (2021).

### Comparison with other DIY WQMs

PortAqua was compared with other DIY WQM based on Arduino boards reported in the literature in the past 5 years (see Table 1). In general, other WQMs incorporate four parameter measurements. These meters require wires attached directly to the microcontroller board or the use of a protoboard which increases the footprint of these devices considerably and does not consider ease of use. While PortAqua is limited to two parameters, these are custom-made and have reduced costs compared to other commercial sensors. A consequence of the use of custom sensors is a higher measurement error in pH measurement (0.4 in PortAqua compared to 0.1 and 0.3 in the others). Although this is not optimal, this error should not be enough to affect comparison with regulations and guidelines which require an error inferior to 0.5. PortAqua also has the advantage of allowing hybrid calibration: it can be calibrated following on-screen guidance using standard buffers for the parameters or it can be calibrated using solutions with non-standard value by directly modifying a text file.

**Table 1:** Comparison between DIY Arduino-based WQMs and PortAqua

Meter	pH		Conductivity		Other sensors	Screen	Arduino board	Area (cm^2^)		Enclosure
	Error	Type	Error	Type				Height	Width	
Alimorong et al. (2020)	2.11%	Commercial	3.72%	Commercial	Temperature Turbidity	No	Mega	10.1^c^	5.3^c^	No
Hong et al. (2021)	NR	Commercial	10%	Commercial	Temperature turbidity	No	Uno	6.9^c^	5.3^c^	No
Osman et al. (2018)	0.1	Commercial	0.3 µS cm^-1^	Commercial	Temperature Turbidity	16x2 Ch. LCD	Uno	6.9^c^	5.3^c^	No
PortAqua (this work)	0.4	Custom	<10%^1^	Custom	None	128x64 px. OLED	Nano	13.2	4.1	3D Printed

*NR* Not reported

^a^measured at 1000 µS cm^-1^

^b^Conductivity measurements used for TDS estimation

^c^measured on Arduino board without sensors

## Conclusion

In this work, we have implemented a modular, compact, lightweight, low-cost, compact, 3D-printed two-parameter (pH and conductivity) WQM called PortAqua. PortAqua effectiveness was evaluated from two perspectives: first in terms of its performance compared to commercial WQMs, and second in terms of the impact on the perception of the water quality measurement of undergraduate and graduate students.

PortAqua capabilities were compared to two commercial WQMs: single-parameter Horiba LAQUATwin pH/conductivity and multi-parameter Hanna HI9829. The cost of the meter components is under $\text {US}\$50$, 20%, and 2% of Horiba and Hanna WQMs, respectively. The pH measurements show a maximum error of 0.4 compared to 0.1 in the commercial WQMs; conductivity measurements are within the range of error of the commercial WQMs at conductivities over the maximum permitted by local regulations. In addition, the meter presents two advantages over commercial WQMs. First, it is not limited to specific calibration standards; it can use any sample with known conductivity for its calibration. Second, as an open-source project, PortAqua offers direct access to the measurement method and allows for validation and modification for anyone interested; this is not possible in low-cost brandless WQMs that offer no independent validation or in close-sourced high-cost systems that limit its modification.

Students were trained to use PortAqua as a technological tool to measure the pH and the conductivity of water samples. The introduction of PortAqua in a hands-on experience evidences the importance of technologies that allow undergraduate and graduate students to classify the local water sources near the campus as drinkable or not according to the local regulations. Pre- and post-workshop assessments revealed a shift from low level of knowledge in all the students to partial or extended knowledge on local water quality regulations, increased understanding of parameters for measuring water quality, and identification of the pH and conductivity maximum values. In this experience, the students also operated and interacted with PortAqua while measuring samples collected from local water sources of the city of *Bogota*. Here, the clear 3D-printed case allowed them to correlate the internal components of the meter with its mechanisms to measure pH and conductivity. After this workshop, the students were eager to share their thoughts and feedback while learning more about PortAqua. These results show that PortAqua is not only a low-cost WQM solution, but also it is a technology that can fill the gap of knowledge about water quality measurement. Moreover, PortAqua could empower communities not only in WQMs, but also in measurement techniques and technology integration. One-to-one real hands-on experience (1 m per user) could be the way to find insights and receive feedback for the development of new modules and future versions based on the community’s needs.

The feedback received from the workshop participants, developers, and general community has resulted in future goals for new PortAqua versions. These goals are:

- Inclusion of new custom, non-commercial modules such as TDS, colorimetric, temperature, and dissolved oxygen.
- Ergonomic improvements and ease of construction.
- Improving the microcontroller board to a Raspberry Pi Pico and shifting the code to MicroPython
- Expand the workshop to bigger communities which currently include the region of La Paz, Cesar - Colombia, which is close to the Cesar Basin mine.

## Data Availability

The datasets generated during and/or analyzed during the current study are available from the corresponding author on reasonable request.
